# Impact of Multimorbidity on Symptom Burden and Symptom Clusters in Patients Receiving Chemotherapy

**DOI:** 10.1002/cam4.70418

**Published:** 2025-02-05

**Authors:** Carolyn Harris, Marilyn J. Hammer, Yvette P. Conley, Steven M. Paul, Bruce A. Cooper, Joosun Shin, Kate Oppegaard, Lisa Morse, Jon D. Levine, Christine Miaskowski

**Affiliations:** ^1^ School of Nursing University of Pittsburgh Pittsburgh Pennsylvania USA; ^2^ Dana‐Farber Cancer Institute Boston Massachusetts USA; ^3^ School of Nursing University of California San Francisco California USA; ^4^ VA Portland Health Care System Portland Oregon USA; ^5^ School of Medicine University of California San Francisco California USA

**Keywords:** cancer, chemotherapy, clusters, multimorbidity, social determinants of health, symptoms

## Abstract

**Background:**

Detailed information on patient characteristics and symptom burden associated with multimorbidity in oncology patients is extremely limited. Purposes were to determine the prevalence of low (≤ 2) and high (≥ 3) multimorbidity in a sample of oncology outpatients (*n* = 1343) undergoing chemotherapy and evaluate for differences between the two multimorbidity groups in demographic and clinical characteristics; the occurrence, severity, and distress of 38 symptoms; and the stability and consistency of symptom clusters.

**Methods:**

Using the Self‐Administered Comorbidity Questionnaire, patients were classified into low and high multimorbidity groups. Memorial Symptom Assessment Scale was used to assess the occurrence, severity, and distress of 38 symptoms prior to the patients' second or third cycle of chemotherapy. For each multimorbidity group, symptom clusters based on occurrence rates were identified using exploratory factor analysis.

**Results:**

Compared to the low group (61.4%), patients in the high group (38.6%) were older, had fewer years of education, were less likely to be married or partnered, less likely to be employed, and had a lower annual income. In addition, they had a higher body mass index, poorer functional status, were a longer time since their cancer diagnosis, and were more likely to have received previous cancer treatments and have metastatic disease. Patients in the low and high groups reported 12.7 (±6.7) and 15.9 (±7.5) concurrent symptoms, respectively. Eight and seven symptom clusters were identified for the low and high groups, respectively. Psychological, gastrointestinal, weight gain, hormonal, and respiratory clusters were stable across multimorbidity groups. Weight gain and respiratory clusters were consistent. Three unstable clusters were identified in the low group and two in the high group.

**Conclusions:**

Findings suggest that higher multimorbidity is associated with various social determinants of health and a higher symptom burden. Differences between multimorbidity groups may be related to aging, treatments, and/or comorbid conditions.

## 1 Introduction

Multimorbidity, defined by the World Health Organization as “the coexistence of two or more chronic conditions in the same individual” (3) [1], presents a major global health challenge as the number of cases increases worldwide [2]. The care of patients with multimorbidity is complicated by the lack of evidence‐based practice guidelines, failure to coordinate care provided by multiple specialists, and polypharmacy [3]. Of note, over 75% of adult oncology patients have at least one chronic comorbid condition at the time of diagnosis [4]. This situation has numerous negative consequences for patients with cancer, including increased length of time to diagnosis [5], increased stage of disease at diagnosis [5], increased likelihood of not receiving curative therapy [6], poorer quality of life [6], and increased mortality [6]. While multimorbidity and cancer are strongly associated with aging [3], the factors associated with the development of both conditions are multifaceted. An increased understanding of the demographic, clinical, social, and behavioral risk factors for multimorbidity in oncology patients will lead to more timely and targeted interventions and increased referrals to supportive care services.

### 1.1 Multimorbidity and Symptom Burden

Only six studies evaluated for associations between multimorbidity and symptom burden in patients with cancer [7, 8, 9, 10, 11, 12]. Of the three studies that used symptom occurrence [7, 10, 12], two reported positive associations between comorbidity burden [10] or the number of chronic conditions [7] and the number of symptoms, while the third found no association [12]. In terms of symptom severity, while in one study [9], no association was found, in another study [8], positive associations were found between the number of comorbid conditions and the number of more severe symptoms (i.e., score ≥ 7). In the sixth study that evaluated symptom distress [11], a higher number of comorbid conditions was associated with higher symptom distress total scores.

These inconsistent findings may be related to differences in the measures of multimorbidity (e.g., Self‐Administered Comorbidity Questionnaire (SCQ), medication review) and symptom burden (e.g., symptom occurrence vs. symptom severity) [7, 8, 9, 10, 11, 12]. In addition, in two studies [7, 11], only older adults were evaluated, and in three studies [8, 10, 12], patients had completed cancer treatment. Of note, only one study evaluated for differences in demographic and clinical characteristics associated with higher levels of multimorbidity and this study was limited to older adults with cancer [7]. Given the paucity of research, the relatively small sample sizes, the inconsistent findings, and the high prevalence of multimorbidity in patients with cancer, a detailed characterization of risk factors for multimorbidity and associations with symptom burden are warranted.

### 1.2 Multimorbidity and Symptom Clusters

Equally important, given the large literature on symptom clusters in patients with cancer [13], only one study evaluated for an association between multimorbidity and symptom cluster factor scores [14]. In this study of patients receiving chemotherapy [14], symptom clusters were identified using Memorial Symptom Assessment Scale (MSAS) severity scores for the total sample. Then, symptom cluster severity scores for each cluster were calculated (i.e., average of the severity ratings for all of the symptoms within the cluster) and categorized into three severity levels (i.e., none = 0, mild = 0 to 1, greater than mild > 1). Next, patients were categorized into multimorbidity groups (i.e., no = 0, low = 1 to 2, high ≥ 3) and differences in symptom cluster severity scores among the multimorbidity groups were evaluated. For the four symptom clusters identified (i.e., nutrition, neurocognitive, abdominal discomfort, respiratory), no significant differences in severity scores were found among the multimorbidity groups. A limitation of this study is that symptom clusters within each of the multimorbidity groups were not evaluated. Given that the stability and consistency of symptom clusters vary by cancer types [15] and across chronic conditions [16], an evaluation of whether clusters vary by multimorbidity status will provide important insights into the symptom burden of oncology patients with multimorbidity. Therefore, in a sample of outpatients (*n* = 1343) with breast, lung, gastrointestinal, or gynecological cancer undergoing chemotherapy, the purposes of this study were to determine the prevalence of low (≤ 2) and high (≥ 3) multimorbidity and evaluate for differences between the two multimorbidity groups in demographic and clinical characteristics; the occurrence, severity, and distress of 38 symptoms; and the stability and consistency of symptom clusters. We hypothesized that patients with high multimorbidity would report higher symptom occurrence, severity, and distress ratings and that symptom clusters would vary by multimorbidity status.

## 2 Methods

### 2.1 Patients and Settings

This analysis was part of a larger study that evaluated symptom clusters in oncology outpatients receiving chemotherapy [15, 17]. Eligible patients were ≥ 18 years of age; had a diagnosis of breast, lung, gastrointestinal, or gynecologic cancer; had received chemotherapy within the preceding 4 weeks; were scheduled to receive at least two additional cycles of chemotherapy; were able to read, write, and understand English; and gave written informed consent. Patients were recruited from two Comprehensive Cancer Centers, one Veteran's Affairs hospital, and four community‐based oncology programs.

### 2.2 Procedures

Eligible patients were approached by a research nurse during their first or second cycle of chemotherapy and provided written informed consent. Patients completed questionnaires six times over two cycles of chemotherapy. Data from the enrollment assessment (i.e., before the patient's second or third cycle of chemotherapy) were used in these analyses. Medical records were reviewed for disease and treatment information. This study was approved by the Committee on Human Research at the University of California, San Francisco, and the Institutional Review Board at each of the study sites.

### 2.3 Instruments

Patients completed a demographic questionnaire, Karnofsky Performance Status (KPS) scale [18], and SCQ [19]. The SCQ consists of 13 common chronic conditions simplified into language that can be understood without prior medical knowledge [19]. Patients indicated if they had the condition, if they received treatment for it (i.e., proxy for disease severity), and if it limited their activities (i.e., indication of functional limitations). For each condition, the patient can receive a maximum of three points. The total SCQ score ranges from 0 to 39. The SCQ has well‐established validity and reliability [20, 21]. Toxicity of each patient's chemotherapy regimen was rated using the MAX2 index [22, 23].

A modified version of the 32‐item MSAS was used to evaluate the occurrence, severity, and distress of 38 common symptoms associated with cancer and its treatment [24]. Six common symptoms were added: hot flashes, chest tightness, difficulty breathing, abdominal cramps, increased appetite, and weight gain. Using the MSAS [24], patients reported whether they had experienced each symptom in the past week. If they had experienced the symptom, they were asked to rate its severity and distress. Severity was measured using a four‐point Likert scale (i.e., 1 = slight, 2 = moderate, 3 = severe, 4 = very severe). Distress was measured using a five‐point Likert scale (i.e., 0 = not at all, 1 = a little bit, 2 = somewhat, 3 = quite a bit, 4 = very much). The validity and reliability of the MSAS are well established [24].

### 2.4 Data Analyses

#### 2.4.1 Creation of Multimorbidity Groups

The number of conditions on the SCQ was totaled for each patient (possible range 1–13). As was done in prior research [7], the number of comorbid conditions was used to dichotomize patients into low (≤ 2) or high (≥ 3) multimorbidity groups. Descriptive statistics and frequency distributions were calculated for the demographic and clinical characteristics, as well as symptom occurrence rates and severity and distress ratings using IBM SPSS Statistics Version 29 (IBM Corporation, Armonk, NY). Differences among the multimorbidity groups in demographic, clinical, and symptom characteristics at enrollment were evaluated using parametric and nonparametric tests.

Exploratory factor analysis (EFA) was used to identify symptom clusters from the occurrence ratings using Mplus Version 8.8 [25]. Separate EFAs were done for each multimorbidity group. Factor loadings were considered meaningful if the loading was ≥ 0.40 [25, 26]. Factors were considered to be adequately defined if at least two items (i.e., symptoms) had loadings of ≥ 0.40 [26]. Items were allowed to load on more than one factor (i.e., cross‐load) if they fell within our preset criteria of ≥ 0.40. Tetrachoric correlations were used to create the matrix of associations for the occurrence items [27]. The simple structure for the EFAs was estimated using the method of unweighted least squares with geomin (i.e., oblique) rotation. The unweighted least‐squares estimator was selected to achieve more reliable results with the dichotomous occurrence items [25].

Factor solutions were estimated for two to eight factors. The factor solution with the greatest interpretability and clinical meaningfulness was selected given that it met the criteria set for evaluating simple structure (i.e., size of item loadings, number of items on a factor). Clusters were named based on the symptoms with the highest factor loadings and the majority of the symptoms within the cluster.

#### 2.4.2 Evaluation of Stability and Consistency

As was done in our previous studies [15, 28], the term stability was used to describe whether or not the same clusters were identified across the two multimorbidity groups. In contrast, consistency was used to describe whether the specific symptoms within a cluster remained the same across multimorbidity groups. For a cluster to be considered consistent, the same two or three symptoms with the highest factor loadings needed to be present across both multimorbidity groups. Given that a symptom cluster must contain a minimum of two symptoms [29], a minimum of the same two symptoms with the highest factor loadings was applied to clusters with only two or three symptoms. For clusters with four or more symptoms, a minimum of the same three symptoms with the highest factor loadings was required to be present across both multimorbidity groups to be considered consistent.

## 3 Results

### 3.1 Demographic and Clinical Characteristics

Of the 1343 patients in this study, 61.4% were in the low (≤ 2 conditions) and 38.6% were in the high (≥ 3 conditions) multimorbidity groups. Differences between the two multimorbidity groups in demographic and clinical characteristics are summarized in Table 1. In brief, patients in the high group were older, had fewer years of education, were less likely to be married or partnered, more likely to self‐identify as Black, less likely to be employed, and had a lower annual income. In addition, they had a higher body mass index (BMI), were more likely to have lung cancer, were a longer time since their cancer diagnosis, had a poorer functional status, and were more likely to have received previous cancer treatments and have metastatic disease. In addition, all of the chronic conditions listed on the SCQ had higher occurrence rates.

**TABLE 1:** Differences in demographic and clinical characteristics between patients with low and high multimorbidity.

Characteristic	Low (≤ 2) Multimorbidity (0)	High (≥ 3) Multimorbidity (1)	Statistics
	61.4% (*n* = 824)	38.6% (*n* = 519)	
	Mean (SD)	Mean (SD)	
Age (years)	54.6 (12.3)	61.3 (11.2)	*t* = −10.28, *p* < 0.001
Education (years)	16.5 (3.0)	15.6 (3.0)	*t* = 5.42, *p* < 0.001
Body mass index (kg/m^2^)	25.6 (5.1)	27.2 (6.4)	*t* = −4.89, *p* < 0.001
Karnofsky Performance Status score	81.9 (11.9)	77.0 (12.9)	*t* = 6.89, *p* < 0.001
Number of comorbidities out of 13	1.5 (0.5)	3.9 (1.1)	*t* = −46.84, *p* < 0.001
Self‐Administered Comorbidity Questionnaire score	3.5 (1.3)	8.6 (2.9)	*t* = −37.37, *p* < 0.001
Alcohol Use Disorder Identification Test score	3.0 (2.4)	2.9 (2.6)	*t* = 1.04, *p* = 0.299
Time since diagnosis (years)	1.6 (3.1)	2.5 (4.8)	U, *p* < 0.001
Time since diagnosis (median)	0.40	0.49	
Number of prior cancer treatments	1.5 (1.5)	1.8 (1.6)	*t* = −3.40, *p* < 0.001
Number of metastatic sites including lymph node involvement	1.1 (1.2)	1.4 (1.3)	*t* = −4.39, *p* < 0.001
Number of metastatic sites excluding lymph node involvement	0.7 (1.0)	1.0 (1.1)	*t* = −4.69, *p* < 0.001
MAX2 score	0.18 (0.08)	0.16 (0.08)	*t* = 5.12, *p* < 0.001
Mean number of MSAS symptoms (out of 38)	12.7 (6.7)	15.9 (7.5)	*t* = −7.91, *p* < 0.001
	% (n)	% (n)	
Gender			FE, *p* = 0.893
Female	77.6 (639)	78.0 (405)	
Male	22.4 (184)	22.0 (114)	
Self‐reported race or ethnicity			*Χ* ^2^ = 12.71, *p* = 0.005
Asian or Pacific Islander	13.4 (110)	11.4 (58)	NS
Black	5.3 (43)	10.2 (52)	0 < 1
Hispanic mixed or others	10.4 (85)	11.2 (57)	NS
White	70.9 (580)	67.1 (341)	NS
Married or partnered (% yes)	68.6 (558)	57.9 (296)	FE, *p* < 0.001
Lives alone (% yes)	18.9 (154)	25.4 (130)	FE, *p* = 0.006
Childcare responsibilities (% yes)	25.5 (206)	16.6 (84)	FE, *p* < 0.001
Care of adult responsibilities (% yes)	6.8 (51)	9.9 (46)	FE, *p* = 0.064
Currently employed (% yes)	41.7 (340)	24.5 (126)	FE, *p* < 0.001
Income			U, *p* < 0.001
< $30,000	11.5 (86)	29.7 (135)	
$30,000 to < $70,000	19.9 (149)	23.1 (105)	
$70,000 to < $100,000	17.9 (134)	15.2 (69)	
≥ $100,000	50.6 (378)	31.9 (145)	
Specific comorbidities (% yes)			
Heart disease	1.3 (11)	12.7 (66)	FE, *p* < 0.001
High blood pressure	13.5 (111)	56.8 (295)	FE, *p* < 0.001
Lung disease	3.0 (25)	24.7 (128)	FE, *p* < 0.001
Diabetes	2.5 (21)	19.5 (101)	FE, *p* < 0.001
Ulcer or stomach disease	1.0 (8)	11.0 (57)	FE, *p* < 0.001
Kidney disease	0.1 (1)	3.5 (18)	FE, *p* < 0.001
Liver disease	1.8 (15)	13.9 (72)	FE, *p* < 0.001
Anemia or blood disease	5.1 (42)	23.5 (122)	FE, *p* < 0.001
Depression	7.9 (65)	37.0 (192)	FE, *p* < 0.001
Osteoarthritis	2.1 (17)	28.3 (147)	FE, *p* < 0.001
Back pain	9.3 (77)	51.8 (269)	FE, *p* < 0.001
Rheumatoid arthritis	0.7 (6)	7.1 (37)	FE, *p* < 0.001
Exercise on a regular basis (% yes)	75.3 (601)	63.7 (328)	FE, *p* < 0.001
Smoking current or history of (% yes)	30.6 (248)	42.9 (219)	FE, *p* < 0.001
Cancer diagnosis			*Χ* ^2^ = 53.13, *p* < 0.001
Breast	44.4 (366)	33.5 (174)	0 > 1
Gastrointestinal	31.8 (262)	28.9 (150)	NS
Gynecological	16.9 (139)	18.1 (94)	NS
Lung	6.9 (57)	19.5 (101)	0 < 1
Type of prior cancer treatment			*Χ* ^2^ = 20.04, *p* < 0.001
No prior treatment	25.9 (208)	23.3 (117)	NS
Only surgery, CTX, or RT	45.5 (365)	36.7 (184)	0 > 1
Surgery and CTX, or surgery and RT, or CTX and RT	17.9 (144)	22.9 (115)	0 < 1
Surgery and CTX and RT	10.7 (86)	17.1 (86)	0 < 1
Metastatic sites			*Χ* ^2^ = 32.28, *p* < 0.001
No metastasis	36.4 (295)	25.9 (133)	0 > 1
Only lymph nodes	24.2 (196)	18.7 (96)	0 > 1
Only nonlymph nodes	18.2 (148)	25.7 (132)	0 < 1
Lymph nodes and other sites	21.2 (172)	29.6 (152)	0 < 1
CTX cycle length			U, *p* < 0.001
14‐day cycle	46.3 (377)	35.4 (181)	
21‐day cycle	47.7 (388)	55.3 (283)	
28‐day cycle	6.0 (49)	9.4 (48)	
Emetogenicity of CTX			U, *p* < 0.001
Minimal/low	15.8 (129)	25.3 (130)	
Moderate	61.7 (502)	60.0 (308)	
High	22.5 (183)	14.6 (75)	
Antiemetic regimens			*Χ* ^2^ = 2.07, *p* = 0.559
None	6.9 (55)	7.4 (37)	
Steroid or serotonin receptor antagonist alone	20.1 (160)	21.0 (105)	
Serotonin receptor antagonist and steroid	46.9 (373)	49.0 (245)	
NK‐1 receptor antagonist and two other antiemetics	26.1 (208)	22.6 (113)	

Abbreviations: CTX, chemotherapy; FE, Fisher's exact test; kg, kilograms; m^2^, meter squared; MSAS, Memorial Symptom Assessment Scale; NK‐1, neurokinin‐1; NS, not significant; RT, radiation therapy; SD, standard deviation; *U*, Mann–Whitney *U* test.

### 3.2 Symptom Occurrence, Severity, and Distress

Table 2 summarizes the differences between the two multimorbidity groups in ratings of symptom occurrence, severity, and distress. In terms of occurrence, 26 of the 38 symptoms (68.4%) had significantly higher occurrence rates in the high group. The 12 symptoms that did not differ between the two groups were hair loss, change in the way food tastes, nausea, constipation, “I don't look like myself,” changes in skin, hot flashes, problems with sexual interest or activity, increased appetite, weight loss, mouth sores, and vomiting. For 25 of the 38 symptoms (65.8%), patients in the high group reported significantly higher severity scores. The 13 symptoms that did not differ between the two groups were nausea, hot flashes, sweats, dizziness, increased appetite, weight gain, itching, abdominal cramps, chest tightness, difficulty swallowing, vomiting, problems with urination, and swelling of the arms or legs. For 19 of the 38 symptoms (50%), patients in the high group reported significantly higher distress ratings. The 19 symptoms that did not differ were change in the way food tastes, nausea, lack of appetite, “I don't look like myself,” feeling nervous, hot flashes, sweats, problems with sexual interest or activity, dizziness, increased appetite, weight loss, weight gain, itching, abdominal cramps, chest tightness, difficulty swallowing, vomiting, problems with urination, and swelling of arms or legs. Differences in the rankings of the occurrence, severity, and distress ratings for the top 10 symptoms between the two multimorbidity groups are listed in Table 3.

**TABLE 2:** Differences in symptom occurrence, severity, and distress between patients with low and high multimorbidity.

Symptoms^a^	Occurrence^b^			Severity^c^					Distress^d^				
	Low (≤ 2)	High (≥ 3)	*p* ^e^	Low (≤ 2)		High (≥ 3)		*p* ^f^	Low (≤ 2)		High (≥ 3)		*p* ^f^
	%	%		Mean	SD	Mean	SD		Mean	SD	Mean	SD	
Lack of energy	80.8	87.0	0.003	1.9	0.7	2.2	0.8	< 0.001	1.6	1.1	2.1	1.2	< 0.001
Difficulty sleeping	66.5	73.2	0.010	1.9	0.8	2.2	0.8	< 0.001	1.7	1.1	1.9	1.1	0.001
Feeling drowsy	55.9	67.2	< 0.001	1.7	0.7	1.8	0.7	0.023	1.0	1.0	1.3	1.1	< 0.001
Hair loss	55.3	54.0	0.651	2.4	1.1	2.7	1.1	0.003	1.8	1.3	2.1	1.4	0.009
Pain	51.5	74.6	< 0.001	1.8	0.7	2.1	0.8	< 0.001	1.6	1.0	2.0	1.1	< 0.001
Changes in the way food tastes	48.6	50.5	0.536	2.1	0.9	2.2	0.9	0.013	1.6	1.2	1.8	1.3	0.116
Worrying	47.9	58.6	< 0.001	1.7	0.7	2.0	0.8	< 0.001	1.4	1.0	1.9	1.1	< 0.001
Difficulty concentrating	47.5	58.8	< 0.001	1.5	0.6	1.6	0.7	0.001	1.3	1.0	1.7	1.1	< 0.001
Numbness/tingling in hands/ft	45.7	62.5	< 0.001	1.8	0.8	1.9	0.9	0.036	1.4	1.1	1.7	1.2	0.002
Nausea	45.7	50.3	0.114	1.7	0.8	1.8	0.9	0.199	1.6	1.1	1.7	1.2	0.305
Feeling sad	41.6	53.0	< 0.001	1.6	0.7	1.8	0.7	0.002	1.4	1.0	1.7	1.1	0.003
Constipation	41.5	46.6	0.070	1.9	0.8	2.1	0.9	< 0.001	1.6	1.2	1.9	1.2	0.005
Dry mouth	39.9	54.0	< 0.001	1.6	0.7	1.9	0.8	< 0.001	1.1	1.0	1.4	1.2	< 0.001
Feeling irritable	38.2	46.2	0.004	1.6	0.7	1.8	0.7	0.003	1.4	1.0	1.6	1.1	0.017
Lack of appetite	38.0	46.6	0.002	1.9	0.8	2.0	0.8	0.032	1.2	1.1	1.4	1.2	0.097
I don't look like myself	37.3	38.6	0.643	2.1	0.9	2.3	1.0	0.022	1.9	1.2	2.1	1.3	0.052
Changes in skin	36.1	36.5	0.907	1.8	0.8	2.1	0.9	< 0.001	1.5	1.1	1.8	1.3	0.015
Feeling nervous	34.0	44.3	< 0.001	1.6	0.6	1.7	0.7	0.038	1.3	0.9	1.5	1.1	0.109
Hot flashes	30.2	34.4	0.116	1.8	0.8	1.9	0.9	0.352	1.4	1.1	1.4	1.2	0.642
Sweats	29.0	34.8	0.029	1.7	0.7	1.9	0.8	0.060	1.3	1.0	1.3	1.2	0.706
Cough	28.4	39.2	< 0.001	1.4	0.6	1.5	0.6	0.039	0.9	1.1	1.2	1.1	0.001
Feeling bloated	28.4	40.6	< 0.001	1.7	0.7	1.9	0.7	0.001	1.4	1.0	1.7	1.1	0.001
Problems with sexual interest/activity	28.1	32.6	0.085	2.3	0.9	2.7	1.1	< 0.001	1.8	1.2	2.0	1.3	0.071
Diarrhea	27.5	32.8	0.042	1.8	0.8	2.0	0.8	0.003	1.3	1.1	1.7	1.2	0.005
Dizziness	25.2	41.0	< 0.001	1.5	0.7	1.5	0.7	0.389	1.2	0.9	1.3	1.0	0.115
Increased appetite	24.1	28.7	0.062	1.7	0.7	1.8	0.7	0.590	0.8	1.1	1.0	1.2	0.123
Weight loss	24.0	27.2	0.195	1.5	0.7	1.7	0.8	0.034	0.9	1.1	1.0	1.2	0.503
Weight gain	22.9	29.3	0.010	1.5	0.7	1.6	0.7	0.115	1.4	1.4	1.4	1.3	0.686
Itching	21.4	30.3	< 0.001	1.7	0.7	1.8	0.8	0.330	1.2	1.0	1.4	1.2	0.368
Mouth sores	19.4	23.3	0.097	1.6	0.7	1.9	0.8	< 0.001	1.3	1.0	1.7	1.1	< 0.001
Shortness of breath	19.3	38.8	< 0.001	1.6	0.7	1.8	0.7	0.003	1.3	1.0	1.7	1.0	< 0.001
Abdominal cramps	18.9	28.2	< 0.001	1.8	0.7	1.9	0.7	0.159	1.6	1.1	1.7	1.1	0.270
Difficulty breathing	14.0	29.3	< 0.001	1.5	0.7	1.8	0.8	0.003	1.4	1.0	1.8	1.2	0.005
Chest tightness	14.0	23.9	< 0.001	1.5	0.6	1.6	0.7	0.089	1.4	1.0	1.4	1.0	0.840
Difficulty swallowing	11.7	17.1	0.007	1.6	0.8	1.8	0.8	0.087	1.6	1.2	1.7	1.1	0.346
Vomiting	11.1	14.4	0.087	1.8	1.0	1.8	0.8	0.754	1.7	1.2	1.7	1.1	0.977
Problems with urination	10.1	20.4	< 0.001	1.7	0.8	1.9	0.8	0.249	1.5	1.2	1.6	1.2	0.536
Swelling of arms/legs	9.8	22.1	< 0.001	1.8	0.8	2.0	0.8	0.328	1.5	1.1	1.7	1.2	0.334

*Note:* Symptom severity and distress scores are for those patients who reported the occurrence of the symptom.

Abbreviation: SD, standard deviation.

^a^ Symptoms are from the Memorial Symptom Assessment Scale with the addition of the following six symptoms: chest tightness, difficulty breathing, increased appetite, hot flashes, abdominal cramps, and weight gain.

^b^ Symptoms are listed in the descending order of occurrence in reference to the low multimorbidity group.

^c^ Severity ratings without zeros: 1 = slight, 2 = moderate, 3 = severe, 4 = very severe.

^d^ Distress ratings: 0 = not at all, 1 = a little bit, 2 = somewhat, 3 = quite a bit, 4 = very much.

^e^ Results using Fisher's exact test.

^f^ Results using the Mann–Whitney *U* test.

**TABLE 3:** Rankings of the top 10 symptoms based on occurrence, severity, and distress between patients with low and high multimorbidity.

	Low (≤ 2) Multimorbidity			High (≥ 3) Multimorbidity		
*Symptom occurrence*						
Rank	Symptom	%		Symptom	%	
1	Lack of energy	80.8		Lack of energy	87.0	
2	Difficulty sleeping	66.5		Pain	74.6	
3	Feeling drowsy	55.9		Difficulty sleeping	73.2	
4	Hair loss	55.3		Feeling drowsy	67.2	
5	Pain	51.5		Numbness/tingling in hands/ft	62.5	
6	Changes in the way food tastes	48.6		Difficulty concentrating	58.8	
7	Worrying	47.9		Worrying	58.6	
8	Difficulty concentrating	47.5		Dry mouth	54.0	
8	—	—		Hair loss	54.0	
9	Nausea	45.7		Feeling sad	53.0	
9	Numbness/tingling in hands/ft	45.7		—	—	
10	Feeling sad	41.6		Changes in the way food tastes	50.5	

*Symptom severity* ^a^						
Rank	Symptom	Mean	SD	Symptom	Mean	SD
1	Hair loss	2.4	1.1	Hair loss	2.7	1.1
1	—	—	—	Problems with sexual interest/activity	2.7	1.1
2	Problems with sexual interest/activity	2.3	0.9	I don't look like myself	2.3	1.0
3	Changes in the way food tastes	2.1	0.9	Changes in the way food tastes	2.2	0.9
3	I don't look like myself	2.1	0.9	Lack of energy	2.2	0.8
3	—	—	—	Difficulty sleeping	2.2	0.8
5	Lack of energy	1.9	0.7	Pain	2.1	0.8
5	Difficulty sleeping	1.9	0.8	Changes in skin	2.1	0.9
5	Constipation	1.9	0.8	Constipation	2.1	0.9
5	Lack of appetite	1.9	0.8	—	—	—
9	Pain	1.8	0.7	Worrying	2.0	0.8
9	Abdominal cramps	1.8	0.7	Lack of appetite	2.0	0.8
9	Changes in skin	1.8	0.8	Diarrhea	2.0	0.8
9	Numbness/tingling in hands/ft	1.8	0.8	Swelling of arms/legs	2.0	0.8
9	Hot flashes	1.8	0.8	—	—	—
9	Diarrhea	1.8	0.8	—	—	—
9	Swelling of arms/legs	1.8	0.8	—	—	—
9	Vomiting	1.8	1.0	—	—	—
10	Feeling drowsy	1.7	0.7	Abdominal cramps	1.9	0.7
10	Itching	1.7	0.7	Feeling bloated	1.9	0.7
10	Increased appetite	1.7	0.7	Mouth sores	1.9	0.8
10	Worrying	1.7	0.7	Dry mouth	1.9	0.8
10	Feeling bloated	1.7	0.7	Sweats	1.9	0.8
10	Sweats	1.7	0.7	Hot flashes	1.9	0.9
10	Nausea	1.7	0.8	Numbness/tingling in hands/ft	1.9	0.9
10	Problems with urination	1.7	0.8	—	—	—

*Symptom distress* ^b^						
Rank	Symptom	Mean	SD	Symptom	Mean	SD
1	I don't look like myself	1.9	1.2	I don't look like myself	2.1	1.3
1	—	—	—	Lack of energy	2.1	1.2
1	—	—	—	Hair loss	2.1	1.4
2	Problems with sexual interest/activity	1.8	1.2	Problems with sexual interest/activity	2.0	1.3
2	Hair loss	1.8	1.3	Pain	2.0	1.1
4	Difficulty sleeping	1.7	1.1	Difficulty sleeping	1.9	1.1
4	Vomiting	1.7	1.2	Worrying	1.9	1.1
	—	—	—	Constipation	1.9	1.2
6	Pain	1.6	1.0	Difficulty breathing	1.8	1.2
6	Abdominal cramps	1.6	1.1	Changes in skin	1.8	1.3
6	Changes in the way food tastes	1.6	1.2	Changes in the way food tastes	1.8	1.3
6	Nausea	1.6	1.1	—	—	—
6	Lack of energy	1.6	1.1	—	—	—
6	Constipation	1.6	1.2	—	—	—
6	Difficulty swallowing	1.6	1.2	—	—	—
10	Changes in skin	1.5	1.1	Shortness of breath	1.7	1.0
10	Swelling of arms/legs	1.5	1.1	Swelling of arms/legs	1.7	1.2
10	Problems with urination	1.5	1.2	Difficulty concentrating	1.7	1.1
10	—	—	—	Difficulty swallowing	1.7	1.1
10	—	—	—	Feeling bloated	1.7	1.1
10	—	—	—	Feeling sad	1.7	1.1
10	—	—	—	Mouth sores	1.7	1.1
10	—	—	—	Vomiting	1.7	1.1
10	—	—	—	Abdominal cramps	1.7	1.1
10	—	—	—	Diarrhea	1.7	1.2
10	—	—	—	Nausea	1.7	1.2
10	—	—	—	Numbness/tingling in hands/ft	1.7	1.2

Abbreviation: SD, standard deviation.

^a^ Severity ratings without zeros: 1 = slight, 2 = moderate, 3 = severe, 4 = very severe.

^b^ Distress ratings: 0 = not at all, 1 = a little bit, 2 = somewhat, 3 = quite a bit, 4 = very much.

### 3.3 Low Multimorbidity Group Symptom Clusters

An eight‐factor solution was selected for the low multimorbidity group's EFA (Table 4). Psychological cluster had five symptoms, and feeling sad, worrying, and feeling nervous had the highest factor loadings. Gastrointestinal cluster had three symptoms, and abdominal cramps and diarrhea had the highest factor loadings. Weight gain cluster had two symptoms, weight gain and increased appetite. Hormonal cluster had two symptoms, sweats and hot flashes. Respiratory cluster had three symptoms, and difficulty breathing and shortness of breath had the highest factor loadings. Nausea and vomiting cluster had two symptoms, vomiting and nausea. Physical and cognitive fatigue cluster had three symptoms, and lack of energy and feeling drowsy had the highest factor loadings. Epithelial cluster had six symptoms, and changes in the way food tastes, lack of appetite, and changes in skin had the highest factor loadings.

**TABLE 4:** Comparison of symptom clusters for patients with low and high multimorbidity^a^.

Symptom cluster	Symptoms	Low (≤ 2) multimorbidity	High (≥ 3) multimorbidity
Psychological	Feeling sad	**0.842**	**0.779**
	Worrying	**0.818**	**0.917**
	Feeling nervous	**0.677**	0.539
	Feeling irritable	0.560	**0.541**
	I don't look like myself	0.441	0.425
	Difficulty sleeping	—	0.486
	Difficulty concentrating	—	0.413
	Total number of symptoms in this cluster	5	7
	Consistency^b^	2/3	
Gastrointestinal	Abdominal cramps	**0.767**	0.418
	Diarrhea	**0.513**	0.402
	Feeling bloated	**0.482**	—
	Vomiting	—	**0.689**
	Lack of appetite	—	**0.654**
	Nausea	—	**0.588**
	Weight loss	—	0.537
	Total number of symptoms in this cluster	3	6
	Consistency^b^	0/3	
Weight gain	Weight gain	**0.958**	**0.971**
	Increased appetite	**0.770**	**0.679**
	Weight loss	—	—0.423
	Total number of symptoms in this cluster	2	3
	Consistency^b^	2/2	
Hormonal	Sweats	**0.878**	0.403
	Hot flashes	**0.830**	**0.431**
	Problems with urination	—	**0.680**
	Total number of symptoms in this cluster	2	3
	Consistency^b^	1/2	
Respiratory	Difficulty breathing	**0.989**	**0.892**
	Shortness of breath	**0.770**	**0.799**
	Chest tightness	0.582	0.547
	Total number of symptoms in this cluster	3	3
	Consistency^b^	2/2	
Nausea and Vomiting	Vomiting	**0.921**	Not identified
	Nausea	**0.579**	
	Total number of symptoms in this cluster	2	
	Consistency^b^	Not evaluated	
Sickness Behavior	Lack of energy	Not identified	**0.697**
	Hot flashes		**0.651**
	Difficulty concentrating		**0.600**
	Feeling drowsy		0.474
	Nausea		0.431
	Feeling nervous		0.414
	Sweats		0.412
	Total number of symptoms in this cluster		7
	Consistency^b^	Not evaluated	
Physical and cognitive fatigue	Lack of energy	**0.855**	Not identified
	Feeling drowsy	**0.621**	
	Difficulty concentrating	0.482	
	Total number of symptoms in this cluster	3	
	Consistency^b^	Not evaluated	
Epithelial	Changes in the way food tastes	**0.725**	Not identified
	Lack of appetite	**0.608**	
	Changes in skin	**0.510**	
	Difficulty swallowing	0.484	
	Weight loss	0.476	
	Hair loss	0.455	
	Total number of symptoms in this cluster	6	
	Consistency^b^	Not evaluated	
Dehydration	Difficulty swallowing	Not identified	**0.612**
	Mouth sores		**0.567**
	Changes in the way food tastes		**0.518**
	I don't look like myself		0.498
	Changes in skin		0.489
	Feeling dizzy		0.443
	Dry mouth		0.440
	Lack of energy		0.417
	Total number of symptoms in this cluster		8
	Consistency^b^	Not evaluated	

*Note:* — = Factor loadings for these symptoms were < 0.40. Bold font indicates symptoms with the highest factor loadings. Not identified = this symptom cluster was not identified for the corresponding multimorbidity group. Not evaluated = consistency was not evaluated because that this symptom cluster was not identified for one of the multimorbidity groups.

^a^ Extraction method: unweighted least squares. Rotation method: Geomin (oblique) rotation.

^b^ Consistency was measured by evaluating whether the same two symptoms (for symptom clusters with a total number of two or three symptoms) or three symptoms (for symptom clusters with a total number of four or more symptoms) with the highest factor loading were present across multimorbidity groups.

### 3.4 High Multimorbidity Group Symptom Clusters

A seven‐factor solution was selected for the high multimorbidity group's EFA (Table 4). Psychological cluster had seven symptoms, and worrying, feeling sad, and feeling irritable had the highest factor loadings. Gastrointestinal cluster had six symptoms, and vomiting, lack of appetite, and nausea had the highest factor loadings. Weight gain cluster had three symptoms, and weight gain and increased appetite had the highest factor loadings. Hormonal cluster had three symptoms, and problems with urination and hot flashes had the highest factor loadings. Respiratory cluster had three symptoms, and difficulty breathing and shortness of breath had the highest factor loadings. Sickness behavior cluster had seven symptoms, and lack of energy, hot flashes, and difficulty concentrating had the highest factor loadings. Dehydration cluster had eight symptoms, and difficulty swallowing, mouth sores, and changes in the way food tastes had the highest factor loadings.

### 3.5 Stability and Consistency

Five stable clusters were identified across the two multimorbidity groups (i.e., psychological, gastrointestinal, weight gain, hormonal, respiratory). In terms of consistency, only the weight gain and respiratory clusters met the criteria for consistency. For the psychological cluster, only two of the three symptoms with the highest factor loadings were consistent across multimorbidity groups. For the gastrointestinal cluster, none of the symptoms with the highest factor loadings were consistent across multimorbidity groups. For the hormonal cluster, only one symptom with the highest factor loading was consistent across multimorbidity groups.

## 4 Discussion

This study is the first to comprehensively evaluate the relationships between multimorbidity, a number of social determinants of health (SDoH, e.g., education, income), and symptom burden in a large sample of patients receiving chemotherapy. In addition, this study is the first to evaluate the stability and consistency of symptom clusters in relationship to multimorbidity. Of note, the prevalence rate of 38.6% for the high group is consistent with an overall worldwide pooled prevalence for multimorbidity of 37.2% [2]. While the total number of symptoms differed between the high (15.9 ± 7.5) and low (12.7 ± 6.7) multimorbidity groups, the mean number of symptoms in both groups was in the moderate range [30]. However, while only 33.6% of the low group met the criteria for a high symptom burden, 47.8% of the high group reported 16 or more concurrent symptoms. This finding supports our a priori hypothesis that higher multimorbidity is associated with an increased symptom burden in oncology patients.

### 4.1 Demographic and Clinical Characteristics

Older age, fewer years of education, not being married or partnered, living alone, not having childcare responsibilities, being unemployed, having a lower income, and race were associated with membership in the high multimorbidity group. Of note, all of these characteristics are considered SDoH that have strong positive associations with multimorbidity [31, 32, 33, 34, 35, 36]. In terms of living arrangements, living with a partner or others provides important structures for financial security, psychological support, and social networks [37]. These relationships encourage healthy behaviors (e.g., smoking cessation, exercise) that guard against the development of age‐related chronic conditions and frailty [38, 39].

Consistent with two systematic reviews [32, 36], having fewer years of education was associated with higher multimorbidity. However, in these same reviews [32, 36], findings were mixed regarding the relationships between income and employment and multimorbidity. The factors that mediate the relationship between education and multimorbidity are multifaceted (e.g., behavioral, economic) and intergenerational [40]. For example, patients with fewer years of education may have lower health literacy and poorer health behaviors. In addition, patients with fewer years of education may face challenges with job security and building a financial safety net throughout their working years, which places these patients at a higher risk for financial toxicity. In a study of patients with metastatic cancer [41], multimorbidity, less than a college education, lower annual household income, and unemployment were associated with financial toxicity. Of note, patients with financial toxicity were over four times more likely to delay filling prescriptions or skip doses and over three times more likely to delay psychosocial or supportive care services [41]. These decisions have a negative impact on cancer prognosis, increased symptom burden, and may contribute to the development of other comorbid conditions following cancer treatment [31]. Given that the factors that increase patients' financial burden vary by lifestage [42], research is needed to develop and test strategies that mitigate financial burden in patients with multimorbidity.

Consistent with a population‐based study of adults aged 30–64 years old without cancer [35], self‐reported Black race was associated with higher multimorbidity. Notably, in that study [35], this relationship persisted after controlling for the level of education, marital status, employment status, and family income, which suggests that multiple factors contribute to this relationship. For example, racial discrimination [43] and/or structural racism [44, 45] may drive the higher occurrence of multimorbidity among Black individuals through a variety of mechanistic pathways (e.g., accelerated biological aging [46, 47]).

In the current study, numerous clinical characteristics were associated with higher multimorbidity (see Table 1). While the mean KPS score for the low group (81.9 ± 11.9) indicates that increased effort is needed to maintain normal functioning [18], for the patients with higher multimorbidity (77.0 ± 12.9), it indicates an inability to work. This decrement in physical functioning may contribute to reductions in exercise and increased BMI that were observed in the high multimorbidity group.

All of the conditions listed in the SCQ were associated with membership in the high multimorbidity group. Notably, the magnitude of these relationships was largest for hypertension (56.8% vs. 13.5%), back pain (51.8% vs. 9.3%), depression (37.0% vs. 7.9%), osteoarthritis (28.3% vs. 2.1%), and lung disease (24.7% vs. 3.0%). These conditions are associated with increased disease burden (e.g., cardiovascular disease) [36, 48], decreased functional status [49], polypharmacy [50], and/or increased mortality [48], which may partially explain why patients in the high multimorbidity group received less toxic and emetogenic chemotherapy. As noted in a systematic review [51], independent of age, comorbidity burden was associated with treatment delays, dose modifications, decreased response rates, and increased mortality. While patients may request dose modifications or forgo treatment, evidenced‐based guidelines designed to support clinician decision‐making regarding cancer treatment in the context of multimorbidity focus on older adults [6, 52]. Given that multimorbidity occurs in 75% of oncology patients [4] and is associated with increases in mortality regardless of age [53], evidenced‐based treatment guidelines that account for multimorbidity warrant development and evaluation.

The findings that patients in the high group had received an increased number and types of cancer treatments and had a higher number of metastatic sites at enrollment may reflect the impact of the patients' comorbid conditions on the cancer. As noted in one review [54], various chronic conditions may promote tumor metastasis and enhance the tumor microenvironment through multiple mechanistic pathways (e.g., chronic inflammation, immune reprogramming). In addition, comorbid conditions may negatively impact treatment efficacy through enhanced cancer metabolism and/or gastrointestinal dysbiosis [54]. However, given that the majority of this research is limited to obesity and diabetes [54], future studies need to determine the influence of other chronic conditions on tumor biology and treatment outcomes.

Consistent with previous research [6, 55, 56], smoking status and lung cancer were associated with higher multimorbidity. Smoking is associated with an increased risk for several chronic diseases, including chronic obstructive pulmonary disease (COPD), type 2 diabetes mellitus (DM), and cardiovascular disease [56], as well as lung cancer [55]. Patients with lung cancer tend to be older, have a history of smoking, be diagnosed at a later stage, and have metastatic disease [55]. Of note, all of these factors were associated with the high multimorbidity group in this study. In contrast, patients with breast cancer were less likely to have high multimorbidity, which may be due to the younger average age of these patients (mean age 53.3 ± 11.6).

### 4.2 Symptom Occurrence, Severity, and Distress

For the majority of the MSAS symptoms, the high group reported significantly higher occurrence (68.4%), severity (65.8%), and distress (50%) ratings. While in a study of older adults [7], no differences were found, in three studies of oncology patients, higher multimorbidity was positively associated with symptom occurrence [10], severity [8], or distress [11]. These findings are not surprising given that the symptoms associated with various chronic conditions continue or exacerbate during cancer treatment. For example, patients with DM report an average of 14.0 concurrent symptoms [57] and patients with human immunodeficiency virus (HIV) report 9.7 [58]. Of note, three of the most common symptoms reported in these two conditions [57, 58] (i.e., lack of energy or tiredness, difficulty sleeping, numbness/tingling in hands/ft) were among the top symptoms reported by the high multimorbidity group. Taken together, this higher symptom burden in the high group may reflect additive or synergistic effects among symptoms across various chronic conditions.

Of note, the 12 symptoms with similar occurrence rates for both multimorbidity groups are “classic” chemotherapy‐ or treatment‐related symptoms (i.e., nausea, vomiting, constipation, changes in the way food tastes, changes in skin, mouth sores, hair loss, “I don't look like myself,” problems with sexual interest or activity, increased appetite, weight loss, hot flashes). This finding suggests that the presence of comorbid conditions does not have a substantial impact on the occurrence of these treatment‐related symptoms. Of note, for seven of these symptoms (i.e., hair loss, changes in the way food tastes, constipation, “I don't look like myself,” changes in skin, problems with sexual interest or activity, weight loss, mouth sores), the severity and/or distress ratings differed significantly between the multimorbidity groups. For the high group, these increases in symptom severity may be attributed to age‐related changes in the epithelium or hair follicles and/or medications associated with various chronic conditions (e.g., xerogenic effects of antihypertensives or antidepressants [59]).

Across the occurrence dimension, four of the top five symptoms were the same between the multimorbidity groups (i.e., lack of energy, pain, difficulty sleeping, feeling drowsy). These symptoms are some of the most common ones reported by oncology patients [7, 12], regardless of age [28, 60] or cancer type [15, 61, 62, 63]. In addition, these symptoms are common in other chronic conditions (e.g., chronic kidney disease [64], DM [65], HIV [58]). These somewhat “ubiquitous” symptoms may arise from common pathophysiological mechanism(s) that occur across chronic conditions. For example, alterations in immune function may explain the high occurrence rates for fatigue across a number of chronic conditions [66].

Top‐ranked symptoms that were unique for the low group were the occurrence of nausea and the severity of vomiting. This finding may be due to the higher proportion of patients in the low group who received highly emetogenic chemotherapy. In contrast, the occurrence of dry mouth and the severity of dry mouth and mouth sores were unique to the high group. These findings may reflect age‐related changes in the oral mucosa and salivary glands [59] as well as the side effects of medications for other comorbid conditions [59].

While 14 of the highest ranked distress scores were common across multimorbidity groups, nine additional symptoms were unique to the high group (i.e., difficulty breathing, shortness of breath, feeling sad, worrying, difficulty concentrating, numbness/tingling in hands/ft, diarrhea, mouth sores, feeling bloated). The respiratory symptoms may be due to the higher rates of lung cancer, lung disease, heart disease, anemia, and smoking in the high multimorbidity group. Patients in the high group had higher rates of self‐reported depression, which may explain the higher distress levels for feeling sad, worrying, and difficulty concentrating. While often attributed to the neurotoxic effects of chemotherapy, numbness/tingling in the hands/feet is common in other chronic conditions (e.g., DM [57], HIV [58]) and may worsen during the patients' cancer treatment. In line with previous research [67, 68], higher levels of distress from diarrhea may be partially explained by the higher prevalence of DM among patients with high multimorbidity. These findings were supported by our previous study that identified distinct chemotherapy‐induced diarrhea (CID) classes [69]. Specifically, patients in the high CID class were more likely to have DM than patients in the none class. In addition, patients in the high CID class were more likely to report mouth sores and feeling bloated.

### 4.3 Symptom Clusters

#### 4.3.1 Psychological Cluster

While first reported in patients with various types of cancer [13, 70], a psychological cluster was found in studies of patients with heart failure [71], HIV [58], and COPD [72]. In contrast to our studies that found this cluster to be stable and consistent across cancer types [15] and in older versus younger patients [28], across the multimorbidity groups, it was not consistent. Of note, feeling sad and worrying had the highest factor loadings in both multimorbidity groups and across studies of patients with cancer [13, 70] and other chronic conditions [58, 73, 74]. While these two symptoms are named differently across studies, they equate with depression and anxiety. Anxiety symptoms are highly prevalent in patients with various chronic conditions, with up to 73% of patients with DM, 75% of patients with COPD, and 49% of patients with heart failure reporting elevated levels of anxiety [75]. In addition, depressive symptoms are reported in 29.8% of patients with hypertension and 60.7% of patients with HIV [58]. Taken together, these findings underscore the importance of routine assessment of these symptoms followed by timely referrals to psychological services for all patients regardless of comorbidity status.

Difficulty sleeping and difficulty concentrating were the other symptoms in psychological cluster for the high multimorbidity group. These two symptoms may be related to other chronic conditions. For example, insomnia, which occurs with hypertension and DM [76], may contribute to problems with concentration and mood disturbance.

#### 4.3.2 Gastrointestinal Cluster

Given that the gastrointestinal cluster is the second most common cluster in studies of patients receiving chemotherapy [13], it is not surprising that this cluster was stable across multimorbidity groups. However, gastrointestinal clusters were identified in other chronic conditions (e.g., chronic kidney disease [77], heart failure [71], HIV [58]), which may explain why the symptoms in this cluster were not consistent across multimorbidity groups. Symptoms unique to the gastrointestinal cluster for the high group were nausea, vomiting, lack of appetite, and weight loss. Of note, these symptoms loaded on the nausea and vomiting and epithelial clusters for the low group, which suggests different etiologies for these symptoms. For example, patients in the high group had higher rates of hypertension and depression compared to the low group and nausea and vomiting are side effects of medications prescribed for these conditions (e.g., beta blockers [78], selective serotonin reuptake inhibitors [79]). In addition, nausea, vomiting, and lack of appetite may be related to the fact that patients in the high group were more likely to have ulcer or stomach disease. Chemotherapy may exacerbate this condition and these symptoms, which may lead to weight loss [80].

#### 4.3.3 Weight Gain Cluster

Given that weight gain and increased appetite had the highest factor loadings, the weight gain cluster was stable and consistent across multimorbidity groups. Of note, these symptoms had the highest factor loadings in a weight change or nutrition cluster in our previous studies of patients with breast [62], gastrointestinal [61], gynecological [63], and lung cancers [81] and in younger versus older adults [28]. While BMI was higher in the high group, in both multimorbidity groups, 29.0% of patients were overweight and 21.6% were obese. This finding suggests that additional research is needed to evaluate the relative contribution of various chronic conditions to patients' nutritional status. Given the negative health effects of being overweight and obese on morbidity and mortality [82], individualized interventions are needed that account for patients' comorbidity burden and functional status.

#### 4.3.4 Hormonal Cluster

While not identified in studies of patients with lung [81] or gastrointestinal [61] cancer, the hormonal cluster was stable in studies of patients with breast [62] or gynecological [63] cancers and in both younger and older adults [28]. Across all of these studies [28, 62, 63], as well as in the current study, sweats and hot flashes had the highest factor loadings. These consistent findings suggest that this cluster may develop due to hormonal changes associated with various cancer treatments and/or aging. Of note, problem with urination was a unique symptom in this cluster in the high group. This finding may be attributed to hormonal imbalances related to aging and/or treatment for cancer and other chronic conditions [83, 84]. For example, urinary incontinence occurs in up to 20% of patients who are obese [85] and in 49% of patients with DM [86]. Of note, in our previous studies of patients with breast and gynecological cancers [62, 63], problem with urination was not included in the evaluation of symptom clusters due to its low occurrence rate.

#### 4.3.5 Respiratory Cluster

Given that some of the most common instruments used to evaluate symptom clusters in patients with cancer (e.g., MD Anderson Symptom Inventory, Edmonton Symptom Assessment Scale) only include one respiratory symptom (i.e., shortness of breath), the respiratory cluster is a recent but consistent cluster found in patients with gynecological [63] and lung [81] cancers, as well as in both younger and older patients with cancer [28]. It is interesting to note that even though the high group had higher rates of smoking, lung disease, and lung cancer, this cluster was stable and consistent across both multimorbidity groups. However, given that the occurrence rates for shortness of breath (38.8% vs. 19.3%), difficulty breathing (29.3% vs. 14.0%), and chest tightness (23.9% vs. 14.0%) were nearly double in the high versus the low group suggests that multimorbidity may contribute to worsening of these symptoms in the high group.

#### 4.3.6 Nausea and Vomiting Cluster

While nausea and vomiting often load on the gastrointestinal cluster [13] as they did for the high group, these symptoms loaded on a separate cluster for the low multimorbidity group. This finding may be partially explained by the fact that these patients had a higher MAX2 score (i.e., indication of receipt of a more toxic chemotherapy regimen), received chemotherapy more frequently, and were prescribed more emetogenic chemotherapy. Of note, despite these factors, the antiemetic regimens did not differ between the groups, which may reflect clinician discretion to provide more intensive antiemetic treatment for patients with higher multimorbidity.

#### 4.3.7 Sickness Behavior Cluster

While the sickness behavior cluster was unique to the high multimorbidity group, the symptoms in this cluster were distributed within other clusters in the low group. Of note, sickness behaviors (e.g., malaise, impaired cognition, nausea) and clusters are common across acute (e.g., influenza virus, corona virus) and chronic (e.g., heart failure, irritable bowel syndrome) conditions. For example, as noted in a systematic review, “vitality” or “weary” clusters were identified in studies of patients with heart failure [71]. In addition, a sickness behavior cluster was identified in several studies of patients with cancer [62, 81, 87, 88, 89, 90]. It is hypothesized that sickness behaviors that occur following an immune stimulus are the result of an evolutionary protective mechanism to conserve energy while the body addresses an infection or tissue injury [91]. Following a cascade of inflammatory events in the periphery, proinflammatory cytokines are produced in the brain, which result in the display of sickness behaviors [91]. Given that inflammatory processes are activated in most chronic conditions (e.g., hypertension, DM, obesity) [92], it is likely that this cluster arises from this common mechanism. However, the inflammatory onslaught may be more pronounced and prolonged in patients with multimorbidity, which may explain why this cluster was identified exclusively in the high multimorbidity group.

#### 4.3.8 Physical and Cognitive Fatigue Cluster

While a physical and cognitive fatigue or malaise cluster was identified in both younger and older patients in two previous studies [28, 60], this cluster was only found in the low multimorbidity group. Of note, the three symptoms that comprised this cluster were three of the symptoms that were found in the sickness behavior cluster in the high group. Given that physical and cognitive fatigue are hallmark symptoms of sickness behaviors [91], it is possible that this cluster may share overlapping mechanisms with the sickness behavior cluster.

#### 4.3.9 Epithelial Cluster

While an epithelial cluster was stable and consistent across younger versus older adults [28], it was not stable across cancer types [15] or in this study, across multimorbidity groups. Only changes in the way food tastes and changes in skin were identified across all of the studies that identified this cluster [28, 61, 62, 81]. The lack of consistency in the symptoms in this cluster may be related to differential effects of various chemotherapy regimens and/or higher doses in the low multimorbidity group.

#### 4.3.10 Dehydration Cluster

While the dehydration cluster includes three symptoms that were found in the epithelial cluster (i.e., difficulty swallowing, changes in the way food tastes, changes in skin), these symptoms warrant consideration as a distinct cluster in the high multimorbidity group. Specifically, feeling dizzy, dry mouth, and lack of energy are common symptoms associated with dehydration. Given that 56.8% of the patients in the high group self‐reported a diagnosis of hypertension, the concurrent use of diuretics or antihypertensives may explain this finding. In addition, difficulty swallowing, mouth sores, and changes in the way food tastes (i.e., symptoms with the highest factor loadings) often occur with dry mouth [59]. Given that dry mouth can occur with other chronic conditions (e.g., DM, kidney disease), disease‐specific medications (e.g., antidepressants, opioids), and polypharmacy [59], the symptoms in this cluster make sense clinically. This finding underscores the importance of evaluating for polypharmacy and multimorbidity regardless of age or cancer type [53].

### 4.4 Limitations

Several limitations warrant consideration. While the SCQ is a valid and reliable measure of comorbidity, it only assesses 13 conditions, includes only one mental health condition, and lacks specific details on a number of chronic conditions. Of note, a recent Delphi consensus study [93] recommended that a minimum of 24 core chronic conditions warrants evaluation in future studies, while an additional 35 conditions should be considered for evaluation. Given the negative consequences of polypharmacy on symptom burden, future research needs to evaluate patients' concomitant medications. In addition, while the MSAS provides a comprehensive assessment of cancer symptoms, it does not include unique symptoms associated with other chronic conditions (e.g., palpitations, orthopnea).

### 4.5 Conclusions

Findings from this study suggest that oncology patients with high multimorbidity experience a higher symptom burden. Notably, many of the risk factors for higher multimorbidity that were identified in this study are SDoH. These findings underscore the need to develop strategies at the institutional and policy levels to address or mitigate these SDoH. For example, as recommended in a recent report by the American Association for Cancer Research [94], healthcare systems need to develop and foster community partnerships. Community engagement and trust can improve cancer screenings in underserved communities as well as inform the development of tailored patient navigation services to support patients with diverse needs throughout cancer care [94]. At the policy level, recommendations included the expansion of healthcare coverage and programs that provide patient transportation to healthcare appointments (e.g., Non‐Emergency Medical Transportation Program, Veterans Transportation Program) [94].

While five symptom clusters were found to be stable across multimorbidity groups (i.e., psychological, gastrointestinal, weight gain, hormonal, respiratory), the symptoms within three of these clusters (i.e., psychological, gastrointestinal, hormonal) were not consistent. In addition, three distinct clusters were identified for the low (i.e., nausea and vomiting, physical and cognitive fatigue, epithelial) and two for the high (i.e., sickness behavior, dehydration) multimorbidity groups. These differences may reflect variability in age‐related changes, treatment intensity, and emetogenicity, as well as comorbid conditions and their treatments. Given these differences, future research needs to examine the mechanisms that underlie this higher symptom burden and develop tailored symptom management interventions. Oncology clinicians need to assess the impact of multimorbidity on patients' symptom burden and collaborate with specialists and supportive care services to manage the complex care needs of these patients.

Furthermore, while aging‐related processes influence the symptom burden of patients with cancer, findings from this study support an emerging literature that suggests that multimorbidity negatively influences patient outcomes across the lifespan [53]. Therefore, research is needed to develop evidenced‐based practice guidelines that will support clinicians' decision‐making processes regarding cancer treatment for patients with multimorbidity.

## Author Contributions

**Carolyn Harris:** conceptualization (lead), data curation (lead), formal analysis (equal), investigation (equal), methodology (lead), resources (equal), software (equal), validation (equal), writing – original draft (equal). **Marilyn J. Hammer:** conceptualization (equal), data curation (equal), investigation (equal), methodology (equal), resources (equal), validation (equal), writing – review and editing (equal). **Yvette P. Conley:** conceptualization (equal), data curation (equal), investigation (equal), methodology (equal), resources (equal), validation (equal), writing – review and editing (equal). **Steven M. Paul:** conceptualization (equal), data curation (equal), formal analysis (equal), investigation (equal), methodology (equal), resources (equal), validation (equal), writing – review and editing (equal). **Bruce A. Cooper:** conceptualization (equal), data curation (equal), formal analysis (lead), investigation (equal), methodology (equal), resources (equal), validation (equal), writing – review and editing (equal). **Joosun Shin:** conceptualization (equal), data curation (equal), investigation (equal), methodology (equal), resources (equal), validation (equal), writing – review and editing (equal). **Kate Oppegaard:** conceptualization (equal), data curation (equal), investigation (equal), methodology (equal), resources (equal), validation (equal), writing – review and editing (equal). **Lisa Morse:** conceptualization (equal), data curation (equal), investigation (equal), methodology (equal), resources (equal), validation (equal), writing – review and editing (equal). **Jon D. Levine:** conceptualization (equal), data curation (equal), investigation (equal), methodology (equal), resources (equal), validation (equal), writing – review and editing (equal). **Christine Miaskowski:** conceptualization (equal), data curation (equal), investigation (equal), methodology (equal), project administration (lead), validation (equal), writing – review and editing (equal).

## Ethics Statement

This study was approved by the Committee on Human Research at the University of California, San Francisco, and by the Institutional Review Board at each of the study sites.

## 5 Conflicts of Interest

The authors declare no conflicts of interest.

## Data Availability

Data are available from the corresponding author following the completion of a data sharing agreement with the University of California, San Francisco.
